# Train-YOLO: An Efficient and Lightweight Network Model for Train Component Damage Detection

**DOI:** 10.3390/s25164953

**Published:** 2025-08-10

**Authors:** Hanqing Zong, Ying Jiang, Xinghuai Huang

**Affiliations:** 1 School of Mechanical and Electronic Engineering, Nanjing Forestry University, Nanjing 210037, China; 2 Shenzhen Research Institute, Southeast University, Shenzhen 518000, China

**Keywords:** rail fault detection, YOLOv8, lightweight network, deep learning

## Abstract

Currently, train component fault detection is predominantly carried out through manual inspection, a process that is inefficient, prone to high omission rates, and carries safety risks. This study proposes an innovative fault detection model for train components based on YOLOv8, aiming to overcome the inefficiencies and high omission rates associated with traditional manual methods. By optimizing the YOLOv8 network architecture and integrating the ADown module, C2f-Rep, and DHD, the model significantly improves computational efficiency and detection accuracy. Experimental results demonstrate that the optimized Train-YOLO model achieves a peak accuracy of 92.9% in train component fault detection. Additionally, it features a smaller model size and reduced computational demands, making it ideal for rapid on-site deployment. A comparison with other leading detection models further highlights the superiority of Train-YOLO in both accuracy and lightweight design.

## 1. Introduction

Rail transport plays a critical role in modern transportation systems due to its efficiency, convenience, speed, and reliability. These factors have significantly contributed to the rapid development of national economies, making railways a cornerstone of modern socioeconomic infrastructure [1]. In 2023, China’s railways handled 3.68 billion passenger trips and transported 3.91 billion tons of freight. The national railway fleet included 21,400 locomotives, 75,800 passenger cars, and 920,000 freight cars. The total operational railway mileage reached 159,000 km, including 45,000 km of high-speed rail, making China a global leader. This robust rail transport system is integral to the nation’s transportation network and has a direct impact on daily life, including essential services like food, shelter, and mobility. Therefore, ensuring the safe and efficient operation of trains is a persistent concern.

Trains often travel long distances, accumulate significant wear [2], and are susceptible to accidents, each of which could lead to severe personal injuries and substantial economic losses [3]. Notable incidents include the 28 April 2008 derailment of train T195 from Beijing to Qingdao, which collided with train 5034, resulting in 70 fatalities and 416 injuries, along with nearly 22 h of disrupted service. In 2011, trains D3115 and D301 collided in another major accident [4]. More recently, on 15 October 2023, the K5133 passenger train from Harbin East to Heihe collided with a derailed engineering vehicle, leading to the derailment of the locomotive and the first four cars. These catastrophic events highlight the critical need for robust train safety measures and have intensified the focus on fault detection in train components.

**Figure 1:**
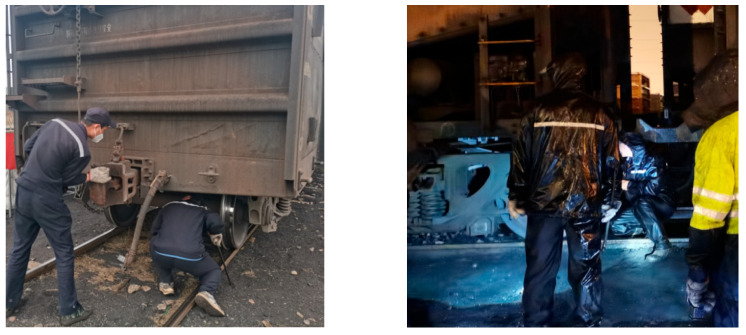
Maintenance workers inspecting a train.

Traditional inspection of train components relies primarily on manual visual examination [5]: inspectors must crawl beneath the vehicle and judge, by eye, whether any component is defective. As shown in Figure 1, maintenance workers are inspecting a train. This procedure is prone to visual fatigue, which leads to inaccurate judgments, a high miss-detection rate, and low efficiency. Moreover, because inspectors work close to the underside of the train, the process involves safety risks [6]. With the rapid development of deep-learning technology, many industries have adopted deep learning to improve inspection efficiency and accuracy [7,8,9]. In railway transportation, the integration of deep learning with inspection technology has become a research hotspot. Chen et al. [10] proposed an adaptive weighted multi-classifier fusion algorithm: multi-direction, multi-channel MFL signals are first classified by SVM [11], weighted according to posterior-probability entropy, and then combined through weighted majority voting to identify rail cracks. Fan et al. [12] presented an SVM model optimized by an improved firefly-optimization algorithm (IFOA) for detecting abnormal rail fasteners; using HOG and LBP features; the method achieves high recognition accuracy on small samples and improves efficiency relative to a conventional SVM. Although these studies have made considerable progress, fault datasets for train components are scarce in practice, data collection is difficult, and deep-learning models are typically large, making deployment on resource-constrained edge devices challenging. Consequently, deep learning is still rarely applied to train-component inspection.

Recently, there has been a surge in deep learning-based research on train detection, with object detection methods becoming the main representative. One approach is based on an end-to-end single-stage algorithm, with the YOLO series models [13] being the primary representatives. For example, Yong et al. [14] addressed switch-machine component detection in complex rail-transit scenarios; by introducing MobileNetV3 and a ResAM attention module into the YOLOv8s [15] backbone and employing Focal IoU Loss to mitigate class imbalance, they developed YOLO-SMPDNet for lightweight detection of key components. Guan et al. [16] proposed a lightweight three-stage railway-obstacle detection framework based on an improved YOLOv4-tiny [17]; by combining multi-scale BING region proposals, IoM region fusion, and residual-attention mechanisms; they built RODNet, which maintains accuracy while reducing parameters and computation, thereby improving deployment efficiency. Zhang et al. [18] developed a MobileNet-YOLOv7-based rail-surface defect detector: MobileNetV3 [19] serves as the backbone to enhance feature extraction, EIOU loss improves localization, and k-means++ optimizes anchor distribution, enabling lightweight defect classification. Chen et al. [20] designed YOLOv8n-FDD, a multi-category fastener-defect detector; CUT-based style transfer generates diverse samples, the CA attention mechanism and an improved loss function enhance feature extraction and generalization, and GSConv [21] plus VoVGSPCP modules yield a lightweight design. Although these approaches are easy to deploy on edge devices and perform well, their localization accuracy is still limited [22]; their performance therefore requires further enhancement and optimization.

Another method is the two-stage algorithm based on object proposal regions. This algorithm can exhibit better accuracy and robustness in more complex scenarios. Choi et al. [23], after collecting 1300 real rail-surface crack images and supplementing them with SEM images of internal defects, used Fast R-CNN [24] to classify and locate rolling-contact-fatigue cracks, achieving higher accuracy than traditional methods. Shang et al. [25] tackled detection of SFC-type fasteners through the Mask-FRCN-based FIQ method: the FRCN module refines segmentation boundaries, the CWD algorithm accelerates inference, and the FCRM method enhances feature extraction, thereby improving detection accuracy. Bai et al. [26] combined an optimized Faster R-CNN with SVDD: improved anchor generation in the RPN boosts detection accuracy, and SVDD further classifies deviated fasteners, reducing misclassification caused by varying orientations. However, because two-stage algorithms involve pixel-level processing, their computational complexity and model size are relatively high, limiting deployment on small edge devices [27].

The YOLO series of models has developed rapidly in the field of object detection [28]. Among them, YOLOv8 is a real-time object detection algorithm that incorporates several improvements compared to its predecessors, significantly enhancing overall performance and flexibility. It offers five different versions: YOLOv8n, YOLOv8s, YOLOv8m, YOLOv8l, and YOLOv8x, designed to suit various usage scenarios and environments. Based on this, the present study develops Train-YOLO, a deep learning model tailored for train component fault detection using YOLOv8. Compared to the original YOLO model, Train-YOLO enhances detection performance while reducing hardware configuration requirements, offering a more efficient and reliable solution for fault detection.

## 2. Dataset Preparation

### 2.1. Data Collection

The original images used in this study’s dataset were collected from a train maintenance company, covering damaged components such as couplers, shock absorbers, sensor valves, and rolling bearing outer races. Since train components undergo strict testing and trials before leaving the factory, the probability of damage during actual operation is extremely low, resulting in a limited number of original damaged samples. To expand the dataset, we further collected images of damaged components accumulated over the past decade from this company and several other train maintenance units. These units maintain a diverse range of train types, including diesel locomotives, electric locomotives, and 10,000-ton heavy-duty trains, with a total operational mileage of nearly 1000 km, providing a representative and diverse sample.

### 2.2. Fault Categorization

**Figure 2:**
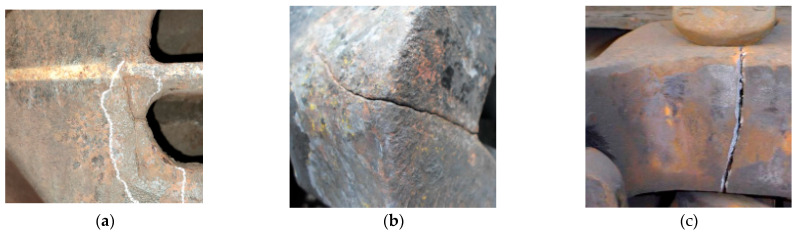
Three types of train component damage: (**a**) fine cracks; (**b**) coarse cracks; (**c**) fractures.

In the actual train maintenance process, maintenance plans need to be differentiated based on the severity of component damage: small cracks are typically repaired by welding, large cracks require a comprehensive evaluation to decide whether to repair or replace, and fractures are usually replaced directly. Therefore, establishing a clear damage classification system is of significant importance. In this study, under the guidance of senior maintenance engineers, the damage was categorized into three types: small cracks, large cracks, and fractures. The engineer team assisted in labeling and reviewing all images to ensure that the category labels were strictly consistent with the real-world conditions. Small cracks and fractures correspond to two standardized maintenance methods, welding and replacement, respectively; large cracks require maintenance personnel to make a comprehensive judgment based on specific conditions. This classification and labeling system significantly reduced the workload of case-by-case decision-making and improved the efficiency and consistency of maintenance decisions. The defect categories in the train component fault dataset are shown in Figure 2.

### 2.3. Data Augmentation

In real-world railway maintenance scenarios, strict safety regulations and the low occurrence rate of faults severely limited image acquisition, leaving us with only 821 original images. Such data scarcity poses two main challenges: first, class imbalance, as severe cracks are significantly rarer than minor ones; second, substantial domain variability due to notable differences in illumination and weather conditions across different maintenance depots.

To alleviate these issues, we employed multiple augmentation techniques, including random flipping, brightness adjustments [29], and the addition of Gaussian noise [30] and salt-and-pepper noise [31]. These augmentations simulated various environmental conditions such as clear weather, snowfall, dappled sunlight, rain, and occlusions, expanding the dataset to 1642 images. Subsequently, all images were re-annotated and verified using the LabelImg tool to ensure labeling consistency.

**Figure 3:**
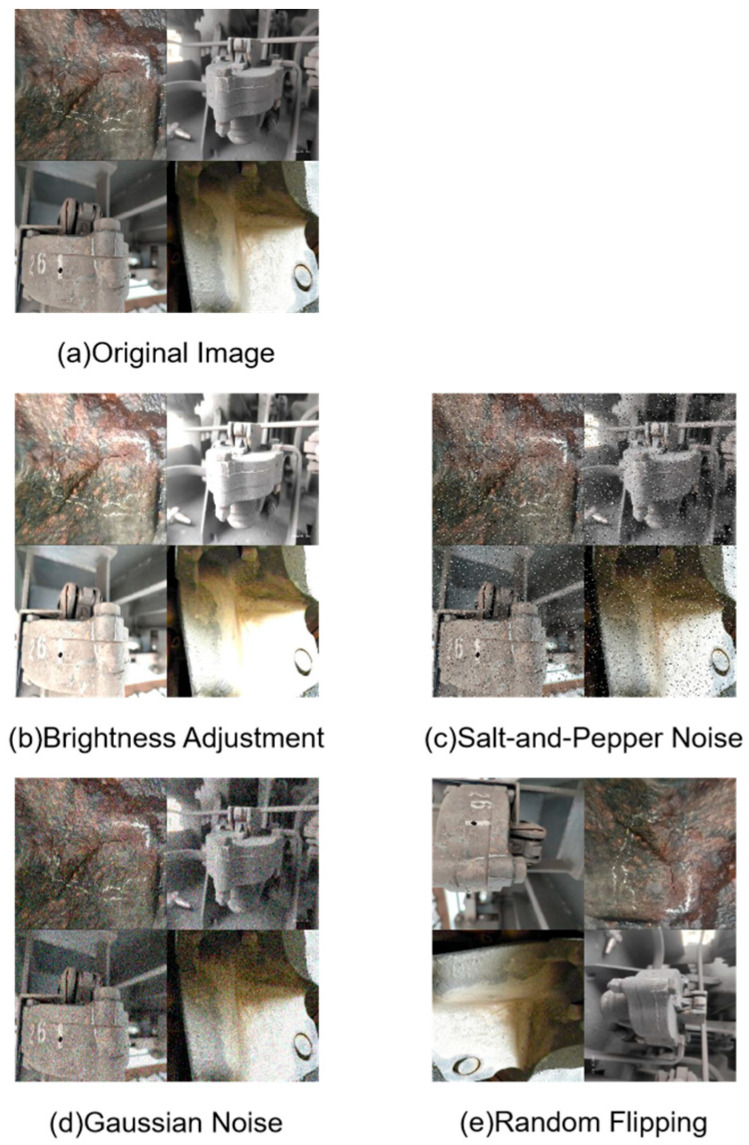
Visual comparison of images before and after data augmentation.

This augmented dataset not only improves the balance among the three severity categories of cracks but also introduces illumination and weather variations closer to real operational conditions, significantly enhancing the generalization capability of the Train-YOLO model. The comparison of the effects before and after data augmentation is exhibited in Figure 3.

In order to better evaluate the model’s performance, the train component fault dataset was randomly divided into training, validation, and test sets in a 7:2:1 ratio, ensuring that the proportions of images and their corresponding labels were similar in each subset. The random division process preserved the diversity and representativeness of both the training and validation sets.

## 3. The Proposed Method Models and Improvements

### 3.1. Original YOLOv8n Model

YOLOv8 integrates the strengths of its predecessors through multiple optimizations. Its backbone network uses an enhanced version of the CSPDarknet53 architecture, combined with YOLOv5’s C2f structure [32], improving the flow of information and gradients. This optimization helps the network capture richer feature information. The neck structure merges the FPN and PAN architectures, facilitating multi-scale feature fusion of images. During the sampling phase, convolutional structures in PAN-FPN [33] are removed, making the model more lightweight and efficient.

**Figure 4:**
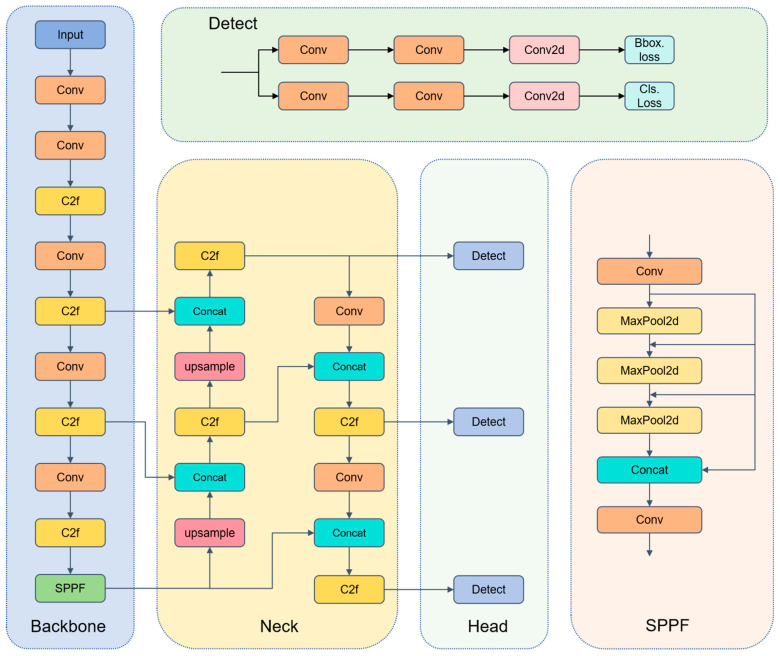
Network architecture diagram of YOLOv8 algorithm.

The head structure adopts a decoupled head design, separating the classification and detection heads and replacing anchor-based methods with anchor-free approaches. This adjustment increases the model’s flexibility and adaptability. The decoupled heads also reduce the number of predicted boxes, speeding up the Non-Maximum Suppression (NMS) process [34]. Additionally, the loss function uses a task-aligned assigner for label matching and employs Distribution Focal Loss [35], further optimizing detection accuracy. Figure 4 illustrates the network architecture of YOLOv8.

### 3.2. Improvement of the YOLOv8 Model: Train-YOLO Model

**Figure 5:**
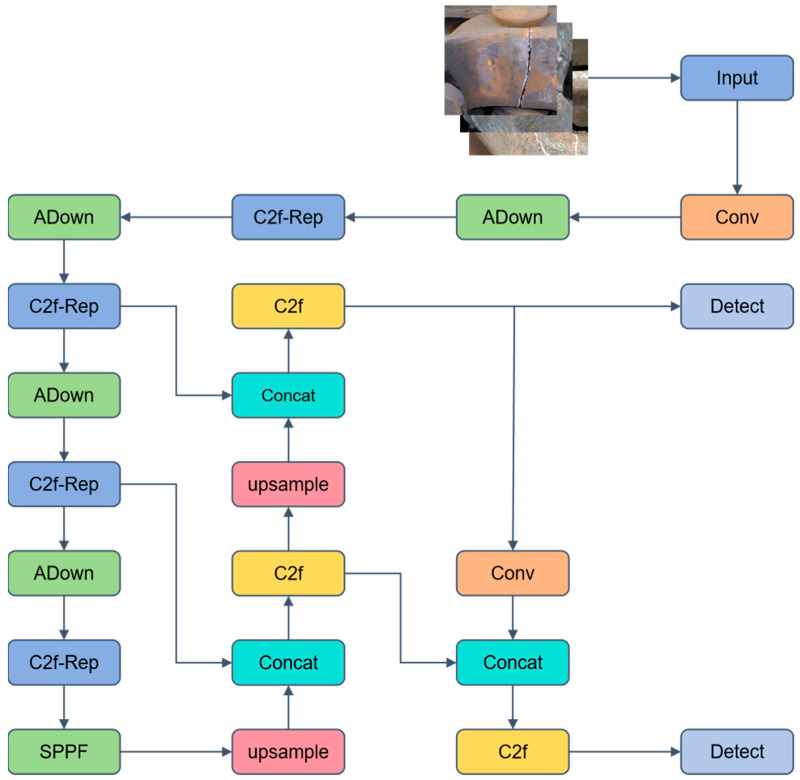
Network architecture diagram of Train-YOLO algorithm.

In train operation and maintenance scenarios, fault detection requires both high detection rates and is constrained by the resources of edge terminals. Therefore, the model must balance both accuracy and lightweight design. To address this dual requirement, this section proposes an improved model based on the advanced YOLOv8 framework, as shown in Figure 5. The detailed improvement strategies are described as follows.

Firstly, ADown [36] is used to replace some of the convolutions. During the input stage, the dual-branch down-sampling effectively suppresses aliasing and retains high-frequency features of small defects, such as fine cracks. This ensures that these small defects can be correctly captured in the subsequent feature extraction process. Secondly, the C2f-Rep is designed to replace the original C2f module. By leveraging cross-layer reuse and structural reparameterization, it enhances the network’s ability to model long-range dependencies, and during the inference phase, it is folded into a single convolution kernel, improving computational efficiency. This significantly enhances the feature representation capability and enables better capture of complex defect characteristics in train components. Finally, the DHD structure is adopted to remove redundant low-resolution detection heads, recovering computational resources and significantly reducing the model’s computational burden. This ensures that the model’s computational efficiency is improved without compromising detection accuracy.

### 3.3. ADown

Defects in train components, particularly fine cracks, are prone to aliasing or blurring during down-sampling. To minimize information loss, we leverage the complementary properties of average pooling and max pooling: the former preserves the overall texture, while the latter emphasizes extreme responses. This approach ensures that defect details are retained to the greatest extent possible while reducing the resolution. Let the input feature map be $X\in R^{c_{1}\times h\times w}$. First, apply average pooling with a stride of 2 to X, yielding

(1) $$\tilde{X}={\mathrm{AvgPool}}_{2}\left(X\right)\in R^{c\times \frac{h}{2}\times \frac{w}{2}}$$

Next, divide $\tilde{X}$ evenly along the channel dimension into two branches.

(2) $$\tilde{X}=\left(X^{\left(a\right)},X^{\left(b\right)}\right),\;X^{\left(a\right)}\in R^{c\times \frac{h}{2}\times \frac{w}{2}},X^{\left(b\right)}\in R^{c\times \frac{h}{2}\times \frac{w}{2}}$$

Branch A: $Y^{\left(a\right)}=Conv_{3\times 3,s=1}\left(X^{\left(a\right)}\right)$, further extracting local spatial patterns;

Branch B: $Y^{\left(b\right)}=Conv_{1\times 1,s=1}\left(MaxPool_{2}\left(X^{\left(b\right)}\right)\right)$, where max pooling is applied first to emphasize peak features, followed by a convolution to adjust the number of channels. The outputs from both branches are then concatenated along the channel dimension to obtain

(3) $$Y=\mathrm{Concat}\left(Y^{\left(a\right)},Y^{\left(b\right)}\right)\in R^{c_{2}\times \frac{h}{2}\times \frac{w}{2}}$$

**Figure 6:**
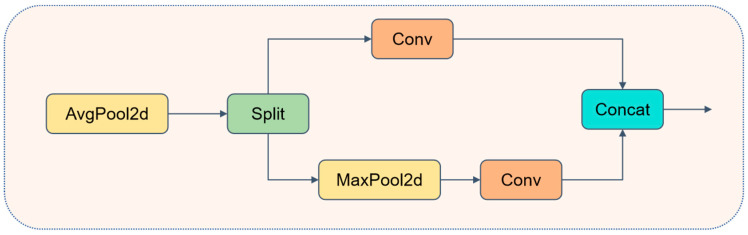
Structure diagram of ADown.

Compared to a single-path 3 × 3 convolution with a stride of 2, ADown reduces the parameter count by approximately 60–70%, while the additional pooling operation introduces almost no extra FLOPs. Through the dual-branch design of “mean aggregation + extreme value enhancement”, ADown effectively suppresses aliasing and preserves sharp defect features, such as cracks, while maintaining a lightweight structure. This design provides higher-quality inputs for subsequent feature fusion modules. As shown in Figure 6, the structure of ADown is illustrated.

### 3.4. C2f-Rep

**Figure 7:**
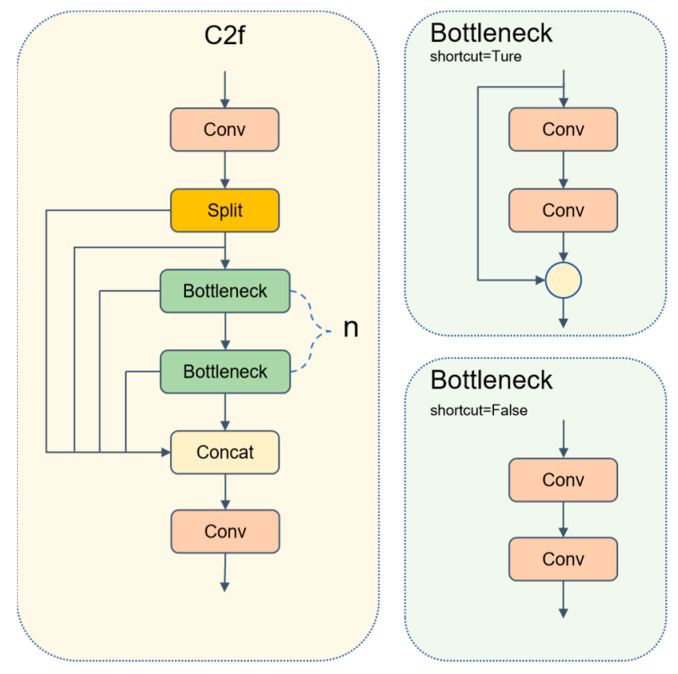
Structure diagram of C2f.

In the task of detecting defects in train components, strip-like cracks and tiny erosion often span multiple receptive fields. Relying solely on single-path convolutions, the network struggles to balance long-range dependencies and fine-grained textures. The C2f module in the YOLOv8 backbone is shown in Figure 7. By adopting a strategy of “channel splitting–multi-Bottleneck stacking–and fusion”, it partially alleviates the above-mentioned issue under the constraint of lightweight design. However, its local convolutions are still insufficient for explicitly modeling long-range context.

To further enhance the representation capability, we propose C2f-Rep. Let the input feature map be $X\in R^{c_{1}\times h\times w}$. We first split X evenly into two branches along the channel dimension.

(4) $$X=\left(X^{\left(a\right)},X^{\left(b\right)}\right),\;\left(X^{\left(a\right)},X^{\left(b\right)}\right)\in R^{c\times \frac{h}{2}\times \frac{w}{2}}$$

The branch $X^{\left(a\right)}$ is fed into the RepViTBlock [37]. This block explicitly separates during the training phase. The token-Mixer (depthwise separable large kernel convolution, used for spatial interaction) and the channel-Mixer (two layers of 1 × 1 convolutions, used for channel reorganization) are employed to capture long-range context. During the inference phase, all branch weights are folded into a single convolution kernel through structural reparameterization.

(5) $$W_{\mathrm{rep}}=\sum_{k=1}^{K}\alpha_{k}W_{k}$$

Here, $\alpha_{k}$ represents the BatchNorm fusion coefficient, and $W_{k}$ denotes the convolution weights of each branch. The other branch, $X^{\left(b\right)}$, undergoes a linear projection through a 1 × 1 convolution. The outputs from both branches are then concatenated along the channel dimension, resulting in

(6) $$Y=\mathrm{Concat}\left(Y^{\left(a\right)},Y^{\left(b\right)}\right)\in R^{c_{2}\times h\times w}$$

**Figure 8:**
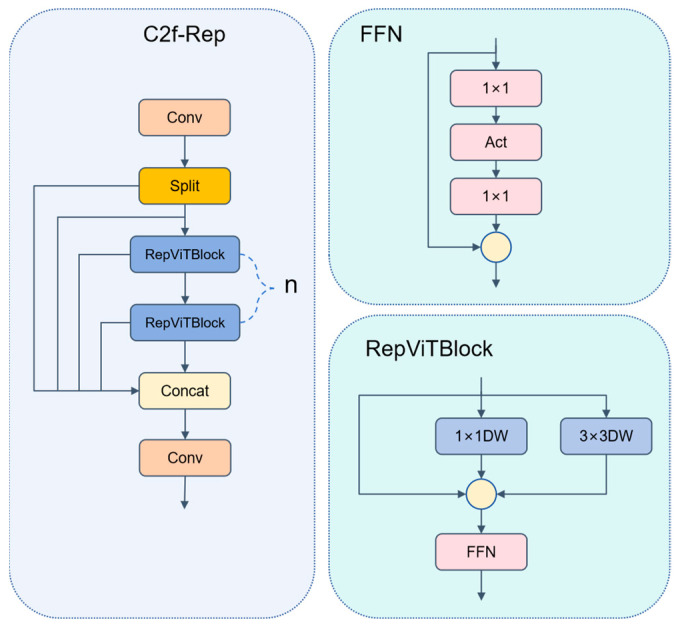
Structure diagram of C2f-Rep.

From Equation (2), it can be observed that during the inference phase, the parameter count and multiply-accumulate operations of C2f-Rep increase linearly with $\;\frac{11}{9}c_{1}c_{2}$, which is significantly lower compared to the traditional C3-Bottleneck ($3\;c_{1}c_{2}$). The cross-layer concatenation helps mitigate gradient decay and enhances the joint representation of fine details and long-range features. Figure 8 summarizes the structural improvements of C2f-Rep compared to C2f.

### 3.5. DHD

In the train component defect detection scenario, most of the targets are of medium-small size. However, the low-resolution detection head $H_{L}$ ($\frac{1}{32}$ feature map) contributes minimally to detecting such targets, yet it occupies about one-third of the detection layer parameters and FLOPs. The native YOLOv8 output contains three sets of detection heads [38].

(7) $$\{H_{S},\;H_{M},\;H_{L}\}=\left\{\frac{1}{8},│\frac{1}{16},│\frac{1}{32}\right\}\times \text{feature map}$$

The three detection heads are responsible for detecting small, medium, and large targets. The Dual-Head Detection (DHD) strategy, based on this, trims the set of detection heads to $\left\{H_{S},\;H_{M}\right\}$. Let the parameter count and computational load of the three-detection-head network be $P_{3},\;F_{3}$, respectively. After pruning:

(8) $$P_{\mathrm{DHD}}=P_{3}-\Delta P,\;\;\;\;\;F_{\mathrm{DHD}}=F_{3}-\Delta F,\;\;\;\;\;\Delta P\approx 0.33P_{3},\;\;\Delta F\approx 0.34F_{3}$$

**Figure 9:**
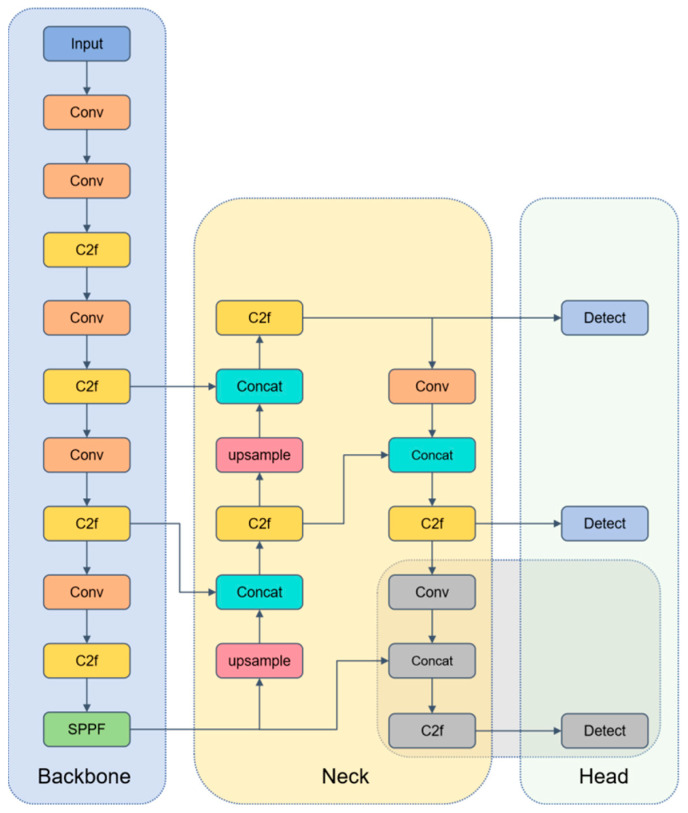
Structure diagram of DHD.

Under the same training configuration, YOLOv8 with the DHD strategy reduces the overall FLOPs by approximately 33% compared to the original three-detection-head network. By reallocating the computational budget from the low-resolution detection head and focusing on high and medium-resolution features, the model achieves improvements in both accuracy and efficiency at the edge, meeting the dual requirements of lightweight design and high precision in train fault diagnosis. Figure 9 shows the structure of DHD.

## 4. Experiments

### 4.1. Experimental Configuration and Training Parameters

The experimental hardware and software conditions are detailed in Table 1 and Table 2.

### 4.2. Evaluation Indicators

We evaluate model performance using the following metrics: Precision (P), Recall (R), Parameters (Params), Floating Point Operations Per Second (FLOP), F1 Score, and Mean Average Precision at IoU threshold of 50% (mAP@50). High mAP@50, recall, and F1 scores indicate the accuracy of the tested model. Small Params and FLOPs suggest a satisfactory real-time model. The formulas are as follows:

Precision

Also known as Positive Predictive Value, it measures the ratio of true positive instances among instances labeled as positive. In train component fault detection, high precision indicates that the model can accurately identify fault instances, reducing the likelihood of misclassifying non-faulty train components as faulty. The calculation formula is given below:

(9) $$\mathrm{Precision}=\frac{\mathrm{TP}}{\mathrm{TP}+\mathrm{FP}}$$

“TP” represents the number of true positives, “FP” represents the number of false positives, and “FN” represents the number of false negatives.

Recall

Also known as Sensitivity or True Positive Rate, it assesses the model’s ability to identify positive instances compared to actual positive instances. In train component damage detection, high recall indicates that the algorithm can accurately identify damage events. The calculation method is as follows:

(10) $$\mathrm{Recall}=\frac{\mathrm{TP}}{\mathrm{TP}+\mathrm{FN}}$$

AP

To evaluate the effectiveness of the model in train component fault detection, it is necessary to consider both recall and precision to ensure effective fault detection and accurate classification simultaneously. Average Precision (AP) provides a comprehensive evaluation of the precision–recall trade-off. The formula for AP is

(11) $$AP=\int_{0}^{1}P\left(r\right)\;dr$$

where the calculation of mAP involves weighting and averaging the average precision values of different categories to obtain mAP:

(12) $$\mathrm{mAP}=\frac{1}{N}\sum_{i=1}^{N}{\mathrm{AP}}_{i}$$

where n represents the total number of categories, and Pi is the average precision of the ith category.

F1 Score

Also known as the harmonic mean, it measures the model’s precision and recall comprehensively. The F1 score ranges from 0 to 1, with 1 indicating optimal model performance. For train component fault detection, the F1 score reflects the model’s ability to precisely identify fault instances while minimizing missed detections. The calculation formula is given below:

(13) $$F1\;=\;2\;\times \;\frac{\text{Precision}\;\times \;\text{Recall}}{\text{Precision}\;+\;\text{Recall}}$$

Params

Represents the total amount of learnable parameters in the neural network, including model weights and biases, used to assess the model’s spatial complexity and size.

GFLOP

Represents the number of billion floating point operations that a model performs per second. GFLOPs are used to evaluate the model’s computational complexity and efficiency.

### 4.3. Ablation Experiments

The Train-YOLO model sequentially integrated DHD, C2f-Rep, and ADown for ablation experiments. These were trained and validated on the dataset, with results shown in Table 3.

The experimental results demonstrate that each enhancement to the Train-YOLO model contributed positively to the final outcomes. Specifically, the following results were recorded:

1. The introduction of the ADown module significantly improved recall and overall detection accuracy while reducing the model size.
2. The implementation of the C2f-Rep module maintained high precision and recall rates while reducing computational demands and model size.
3. The incorporation of the DHD structure dramatically reduced parameters to 1.67 and significantly enhanced precision, as well as reducing model size.

When the three modules work in collaboration, a highly effective synergistic optimization mechanism is formed through hierarchical feature processing, long-range semantic modeling, and the recovery of computational resources. This mechanism not only improves the detection accuracy of the model but also significantly reduces the computational overhead. As a result, the Train-YOLO model becomes more lightweight, with significantly reduced computational requirements and model size, making it particularly suitable for deployment on railway field equipment for detecting train component damage. This meets the practical needs of the railway industry for rapid and efficient fault detection.

### 4.4. Comparative Experiments

To further verify the reliability of the Train-YOLO model in detecting train component faults and to demonstrate its superiority over current state-of-the-art models, we conducted comparative experiments using mAP@0.5, Recall, Precision, F1 Score, and Size/MB as evaluation metrics. Under the same experimental conditions, our Train-YOLO model was compared with Faster-RCNN, SSD [39], YOLOv5, NanoDet [40], EfficientDet-Lite [41], and YOLOv3 models.

Based on the experimental results summarized in Table 4, Train-YOLO outperforms all other models in terms of key performance metrics. Specifically, Train-YOLO achieves the highest Precision (P = 0.929) and F1 Score (F1 = 0.852), indicating its superior ability to accurately detect faults while maintaining a balanced performance across both precision and recall. While YOLOv3 and YOLOv8 show strong results with high precision and mAP@50, Train-YOLO surpasses both in precision, recall, and overall F1 score, suggesting that the model is more robust for fault detection in train components. Notably, Train-YOLO achieves a lower model size (2.90 MB) compared to YOLOv3 and YOLOv8, making it much more efficient for edge deployment without sacrificing detection accuracy. In comparison to models like SSD, Faster RCNN, and EfficientDet-Lite, Train-YOLO significantly outperforms in terms of both detection performance and efficiency, particularly when considering its reduced size and improved F1 score. Although YOLOv5 and NanoDet are also lightweight models, Train-YOLO leads in overall performance metrics, especially in recall and F1 score. In conclusion, Train-YOLO offers the best combination of high detection accuracy, recall, and efficiency, making it a highly suitable solution for real-time train component fault detection on edge devices.

Table 5 presents a comparison of the detection performance of Train-YOLO and YOLOv8 across three different fault types: fine cracks, coarse cracks, and fractures. The performance metrics included in the table are Precision (P), Recall (R), and mAP50 (mean Average Precision at 50 IoU), which are used to evaluate the model’s detection accuracy, recall capability, and overall precision.

The comparison results in the table show that Train-YOLO outperforms YOLOv8 in terms of Precision, Recall, and mAP across all three fault types (fine cracks, coarse cracks, and fractures). This indicates that Train-YOLO offers higher accuracy and better overall performance in train component fault detection, making it more suitable for practical applications, especially in scenarios that require higher precision.

**Figure 10:**
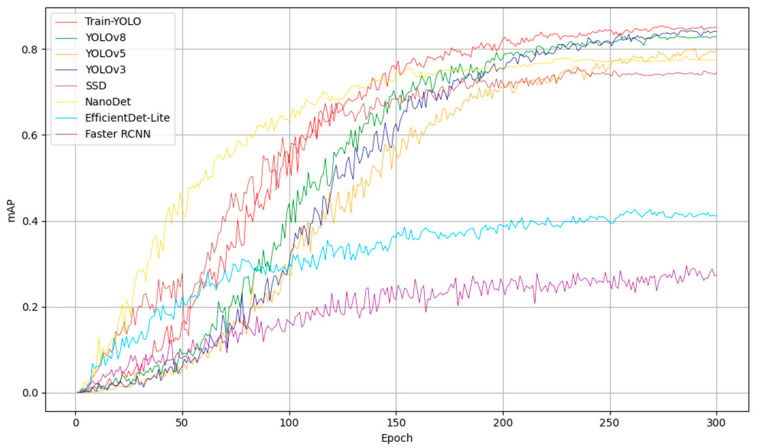
Comparison of the mAP@0.5 curves between Train-YOLO and other models.

The comparison of mAP@0.5 between YOLOv8 and Train-YOLO is shown in Figure 10. As seen from the figure, the mAP–epoch curves reveal three decisive advantages of Train-YOLO over the reference detectors. First, the navy curve crosses the pragmatic 0.60 mAP threshold at epoch 109—approximately 20 epochs earlier than YOLOv8 and approximately 48 epochs earlier than YOLOv5—indicating a markedly faster transition into the useful-accuracy regime. Second, the steeper ascent between epochs 50 and 140 and the eventual plateau at approximately 0.85 mAP (versus approximately 0.83 for YOLOv8 and approximately 0.79 for YOLOv5) confirm both superior learning efficiency and a higher asymptotic accuracy. Third, post-convergence fluctuations are minimal, evidencing a more stable optimization trajectory than the competing models. Crucially, these gains are achieved with a parameter count and FLOPs budget comparable to the ultra-light YOLOv8-nano, yielding the best accuracy-per-computation ratio among all evaluated detectors. Together, these data trends demonstrate that the proposed module blend accelerates learning, raises the ultimate accuracy ceiling, and maintains stable optimization, making Train-YOLO especially attractive for resource-constrained railway defect-inspection tasks.

## 5. Discussion

This paper evaluates the detection performance of the Train-YOLO model using a train component fault dataset, which has been augmented to include instances of train component faults across various scenarios, forming a comprehensive fault dataset. The experiments integrate the YOLOv8 model with various data augmentation techniques to assess its performance improvements.

The evaluation process includes a thorough examination of the detection accuracy and lightweight characteristics of the Train-YOLO model. A comparative analysis was conducted, pitting the Train-YOLO model against other existing models, with evaluation criteria that not only included detection accuracy but also lightweight metrics to fully understand the model’s performance.

These experiments aim to objectively demonstrate the superiority of the Train-YOLO model in terms of detection performance and lightweight characteristics compared to other models. Utilizing the train component fault dataset, we are able to robustly assess the model’s ability to recognize train component faults under various conditions and scenarios.

## 6. Conclusions

In response to the inefficiencies and high rates of oversight associated with manual observations in preliminary train maintenance, this paper presents an innovative train fault detection model based on YOLOv8. To reduce computational complexity, various methods were employed to optimize the YOLOv8 network structure, replacing the neck’s C2f with C2f-Rep and Conv with ADown, addressing hardware resource and computational limitations to facilitate rapid deployment in train fault detection devices. Additionally, the implementation of DHD operations eliminated the YOLOv8’s P5 detection head, further reducing computational demand. Experimental results demonstrate that the proposed method possesses efficient learning capabilities and high recognition accuracy, achieving a peak detection accuracy of 92.9%. The performance and size of the detection model are also superior to those of other mainstream network models. Ablation experiments show that each improvement effectively enhances the algorithm’s performance. Comparative experiments with other mainstream network models reveal that the proposed method achieves a balance between precision and model size, providing a high-precision and efficient solution for train fault detection. Due to the limited size of the experimental dataset, future work could further explore the model’s scalability and generalizability to better adapt to different environments and scenarios.

## Figures and Tables

**Table 1:** Experimental hardware l environment.

Device	Configuration
CPU	AMD Ryzen 9 7945HX
GPU	NVIDIA GeForce RTX 4060
System	Windows 11
Framework	Pytorch 2.2.2
IDE	Pycharm 2023.2.2
Python version	version 3.11.8

**Table 2:** Experimental parameter settings.

Parameter	Setting
Input image size	640 × 640
Epochs	300
Batch size	8
Initial learning rate	0.01
Optimizer	SGD
Python version	version 3.11.8

**Table 3:** Ablation experiment results.

Model	Params/M	GFLOPs	P/%	Recall/%	mAP@50/%	F1 Score	Size/MB
YOLOv8	3.01	8.2	87.8	76.5	82.7	81.7	5.98
YOLOv8 + ADown	2.71 (−0.30)	7.5 (−0.7)	87.1 (−0.7)	79.2 (+2.7)	85.8 (+3.1)	83.0 (+1.3)	5.44 (−0.54)
YOLOv8 + C2f-Rep	2.68 (−0.33)	7.3 (−0.9)	86.3 (+1.5)	77.9 (+1.4)	81.9 (−0.8)	81.9 (+0.2)	5.39 (−0.59)
YOLOv8 + DHD	2.00 (−1.01)	7.3 (−0.9)	92.9 (+5.1)	74.1 (−2.4)	84.0 (+1.3)	82.4 (+0.7)	4.01 (−1.97)
YOLOv8 + DHD + C2f-Rep	1.67 (−1.34)	6.5 (−1.7)	84.5 (−3.3)	76.7 (+0.2)	81.9 (−0.8)	80.4 (−1.3)	3.43 (−2.55)
YOLOv8 + DHD + ADown	1.71 (−1.30)	6.7 (−1.5)	89.3 (+1.5)	77.3 (+0.8)	84.6 (+1.9)	82.9 (+1.2)	3.48 (−2.50)
YOLOv8 + Adown + C2f-Rep	2.40 (−0.61)	6.6 (−1.6)	87.8 (0.0)	78.9 (+2.4)	83.6 (+0.9)	83.1 (+1.4)	4.86 (−1.12)
Train-YOLO	1.38 (−1.63)	5.8 (−2.4)	92.9 (+5.1)	78.6 (+2.1)	84.9 (+2.2)	85.2 (+3.5)	2.90 (−3.08)

**Table 4:** Comparison of experimental results for different network models.

Model	P	Recall	mAP@50	F1 Score	Size/MB
SSD	0.79	0.402	0.743	0.533	91.6
Faster RCNN	0.515	0.811	0.281	0.630	108
YOLOv3	0.915	0.768	0.851	0.835	207.8
YOLOv5	0.824	0.727	0.800	0.772	5.3
YOLOv8	0.878	0.765	0.827	0.817	5.98
NanoDet	0.834	0.585	0.775	0.688	16.2
EfficientDet-Lite	0.426	0.382	0.411	0.403	12.0
Train-YOLO	0.929	0.786	0.849	0.852	2.90

**Table 5:** Comparison of Fault-Detection Performance between Train-YOLO and YOLOv8.

Fault Type	P		R		mAP	
	Train-YOLO	YOLOv8	Train-YOLO	YOLOv8	Train-YOLO	YOLOv8
Fine cracks	0.932	0.875	0.655	0.628	0.764	0.729
Coarse cracks	0.934	0.916	0.812	0.757	0.856	0.818
Fractures	0.923	0.843	0.889	0.911	0.927	0.934

## Data Availability

The data presented in this study are available on request from the corresponding author. The data are not publicly available because the data involve sensitive information.
